# Optimising treatment for COPD – new strategies for combination therapy

**DOI:** 10.1111/j.1742-1241.2009.02139.x

**Published:** 2009-08

**Authors:** T Welte

**Affiliations:** Department of Respiratory Medicine, Hannover Medical SchoolHannover, Germany

## Abstract

Chronic obstructive pulmonary disease (COPD) is a multi-component disease characterised by airflow limitation and airway inflammation. Exacerbations of COPD have a considerable impact on the quality of life, daily activities and general well-being of patients and are a great burden on the health system. Thus, the aims of COPD management include not only relieving symptoms and preventing disease progression but also preventing and treating exacerbations. Attention towards the day-to-day burden of the disease is also required in light of evidence that suggests COPD may be variable throughout the day with morning being the time when symptoms are most severe and patients’ ability to perform regular morning activities the most problematic. While available therapies improve clinical symptoms and decrease airway inflammation, they do not unequivocally slow long-term progression or address all disease components. With the burden of COPD continuing to increase, research into new and improved treatment strategies to optimise pharmacotherapy is ongoing – in particular, combination therapies, with a view to their complementary modes of action enabling multiple components of the disease to be addressed. Evidence from recent clinical trials indicates that triple therapy, combining an anticholinergic with an inhaled corticosteroid and a long-acting β_2_-agonist, may provide clinical benefits additional to those associated with each treatment alone in patients with more severe COPD. This article reviews the evidence for treatment strategies used in COPD with a focus on combination therapies and introduces the 3-month CLIMB study (Evaluation of Efficacy and Safety of Symbicort as an Add-on Treatment to Spiriva in Patients With Severe COPD) which investigated the potential treatment benefits of combining tiotropium with budesonide/formoterol in patients with COPD with regard to lung function, exacerbations, symptoms and morning activities.

**Review Criteria**

A comprehensive web-based literature review focusing on clinical trials and reviews discussing current and future combination therapeutic management approaches for patients with COPD.

**Message for the Clinic**

Chronic obstructive pulmonary disease is a multi-component disease; for optimal treatment, combining therapies with complementary modes of action enables multiple components of the disease to be treated. Evidence suggests that triple therapy combining inhaled corticosteroids and long-acting β_2_-agonists with tiotropium may provide further clinical benefits and may improve quality of life as a result of effects of the complementary modes of action in maintaining airway calibre. The CLIMB study is investigating the treatment benefits of budesonide/formoterol added to tiotropium on a range of important aspects including lung function, morning symptoms and activities and exacerbations.

## Introduction

Chronic obstructive pulmonary disease (COPD) is one of the principal causes of death in most countries and its prevalence is increasing. It is a multi-component disease characterised by airflow limitation in the lungs that is generally progressive and not fully reversible (1). While the presence and severity of symptoms can follow a variable course, characteristic symptoms include chronic cough, sputum production and progressive dyspnoea (1). Acute exacerbations, characterised by physiological deterioration and an increase in airway inflammation, are common, particularly in the later stages of the disease, and have a considerable impact on quality of life, daily activities and general well-being (1–4). Acute exacerbations, in particular, are indicators of a poor prognosis, with mortality risk increasing with the frequency of severe exacerbations (5). Evidence suggests that COPD may be variable throughout the day, with symptoms and activity limitation being most problematic in the mornings (6). In patients with severe COPD, shortness of breath is reported to be the most frequently reported symptom, impacting greatly on morning routines and daily life (7).

Currently, there are several recommended classes of therapy for COPD, of which bronchodilators (including β_2_-agonists and anticholinergics) are the mainstay of symptom management in mild and moderate disease, prescribed on an as-needed basis for mild COPD and as a maintenance therapy for moderate COPD (1). For the treatment of more severe COPD, guidelines recommend the addition of inhaled corticosteroids (ICSs) to long-acting bronchodilator therapy (1). Combinations of therapies have been investigated with a view to their complementary modes of action enabling multiple components of the disease to be addressed. Data from recent clinical trials indicate that triple therapy, combining an anticholinergic with an ICS and a long-acting β_2_-agonist (LABA), may provide clinical benefits additional to those associated with each treatment alone in patients with moderate to severe COPD (8–11). This article reviews the evidence for treatment strategies used in COPD with a focus on combination therapies.

## Guidelines for COPD management

The objectives of COPD management, as defined by the Global Initiative for Chronic Obstructive Lung Disease (GOLD) guidelines, are to relieve symptoms, prevent disease progression, improve exercise tolerance and health status, prevent and treat complications and exacerbations and reduce mortality (1). The poor prognosis and mortality associated with exacerbations of COPD mean that reducing the frequency and severity of exacerbations is one of the central components of a management plan.

**Figure 1:**
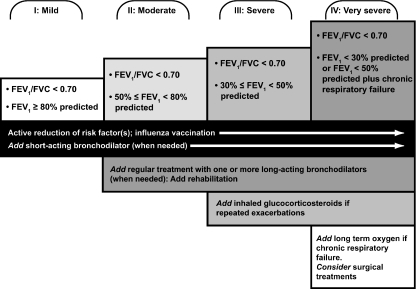
Therapy at each stage of chronic obstructive pulmonary disease (COPD), as recommended by Global Initiative for Chronic Obstructive Lung Disease (GOLD) guidelines (1)

As COPD is a progressive disease, guidelines including those of GOLD (1), the American Thoracic Society/European Respiratory Society (12) and the National Institute for Health and Clinical Excellence (13) recommend a stepwise approach to treatment (Figure 1). Stages of severity are defined according to symptoms and lung function and therapy is tailored to the individual according to their disease stage and treatment response. Bronchodilators are central to the symptomatic management of COPD. Their ability to relax smooth muscle cells in the airway allows improved airflow, indicated by improvements in spirometric parameters and reduced breathlessness (1,14). While bronchodilators do not modify the decline of lung function in COPD, evidence does suggest they can reduce the rate of exacerbations compared with placebo (15,16) and improve dyspnoea, quality of life and exercise capacity (17–19). In the early stages of COPD (mild; stage I), short-acting inhaled bronchodilators are the recommended first-line therapy, to be used on an as-needed basis for rapid control of symptoms. These agents have a fast onset of action but require multiple daily dosing, with the choice of agent depending on availability and on the patient’s response in terms of symptom relief and side effects. Short-acting β_2_-agonists have a bronchodilatory effect that lasts for 4–6 h, while short-acting anticholinergics last up to 9 h (1). For patients with moderate (stage II) to very severe (stage IV) COPD whose dyspnoea is not controlled with short-acting bronchodilators, adding regular maintenance treatment with long-acting inhaled bronchodilators is recommended. The short-acting bronchodilator may still be used as needed if required. Long-acting bronchodilators, including LABAs such as formoterol or salmeterol, and long-acting anticholinergics such as tiotropium can be more effective than short-acting agents and provide sustained improvements (20–22). Slow-release preparations of theophylline have efficacy in COPD and may possess an anti-inflammatory effect (23,24), but the bronchodilatory effect is small and inferior to that of LABAs (25) and the agent is associated with potential toxicities. Consequently, theophylline is only recommended when inhaled bronchodilators are not available (1). When symptoms are not controlled by one class of bronchodilator, combination treatment with the alternative class is recommended and may improve efficacy and decrease the risk of side effects (1). For patients with COPD stages III–IV and a history of repeated exacerbations, guidelines recommend adding an ICS to long-acting bronchodilator therapy (1). Two ICS/LABA combinations have received regulatory approval for COPD: budesonide/formoterol (Symbicort®; AstraZeneca, Lund, Sweden) and salmeterol/fluticasone (Seretide™; GlaxoSmithKline, Greenford, UK).

## Evidence supporting use of long-acting bronchodilators in COPD

### Long-acting anticholinergics

**Figure 2:**
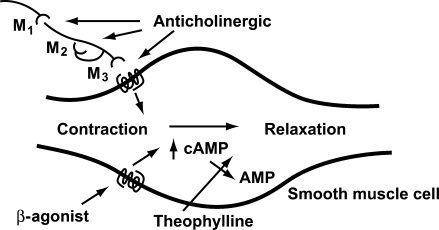
Mechanisms of action of bronchodilators on airway smooth muscle (14). AMP, adenoside monophosphate; cAMP, cyclic adenosine monophosphate; M_1_, M_2_ and M_3_ are muscarinic receptors. Reprinted from Ref. (14). Copyright^©^ (2007)

Anticholinergics act by blocking the effect of acetylcholine on muscarinic receptors (Figure 2). The long-acting inhaled anticholinergic tiotropium is one of the most widely prescribed agents for COPD and is recommended as maintenance therapy for patients with COPD of at least moderate severity. Tiotropium disassociates slowly from the M_1_ and M_3_ muscarinic receptors, providing bronchodilation for more than 24 h and thus making it suitable for once-daily administration (26). Evidence supports significant improvements in bronchodilation and lung function vs. placebo with once-daily dosing that are sustained for 24 h according to measurements of trough forced expiratory volume in 1 s (FEV_1_) 1 h predose, vital capacity and inspiratory capacity (27–30). However, the recent Understanding the Potential Long-term Impacts on Function with Tiotropium (UPLIFT) study – a randomised, double-blind trial comparing 4 years of therapy with either tiotropium or placebo in almost 6000 patients – showed that mean improvements in FEV_1_ with tiotropium (ranging 87–103 ml before bronchodilation and 47–65 ml after bronchodilation) were significantly better than placebo at all time points after randomisation (p < 0.001), although the differences in mean decline in FEV_1_ were not statistically significant (8). The authors suggest this finding may be related to the protocol permitting patients to continue using previously prescribed medications other than inhaled anticholinergics, e.g. ICS and LABA, with the high rate of prescriptions for concomitant respiratory medications in both arms of the study (Table 1; 8).

**Table 1:** Respiratory medications used at baseline in UPLIFT (8)

Medication use (%)	Tiotropium (*n* = 2986)	Placebo (*n* = 3006)
**Any**	93.4	93.1
Inhaled anticholinergic*		
Short-acting	44.9	44.1
Long-acting	2.0	1.6
**Inhaled β**_**2**_-agonist*		
Short-acting	68.5	68.1
Long-acting	60.1	60.1
**Corticosteroid**		
Inhaled*	61.6	61.9
Oral	8.4	8.3
**Theophylline compound**	28.4	28.5
**Mucolytic agent**	7.4	6.9
**Leucotriene-receptor antagonist**	3.3	3.1
**Supplemental oxygen**	2.3	1.9

* This medication was used either alone or as a fixed combination. UPLIFT, Understanding the Potential Long-term Impacts on Function with Tiotropium.

Anticholinergic agents are poorly absorbed systemically, which limits systemic adverse effects. The most frequent side effect reported in clinical trials is dryness of the mouth and more serious effects appear to be rare (31). There are recent reports of elevations in cardiovascular risk, although these findings have not been fully elucidated and this risk remains uncertain. While a retrospective database analysis showed an increased risk of mortality with the inhaled anticholinergic ipratropium [odds ratio 1.34; 95% confidence interval (CI): 1.22–1.47] (32) and a meta-analysis of five long-term trials involving more than 7000 patients found an elevated risk of cardiovascular events and death with both ipratropium [relative risk (RR) 1.57, 95% CI: 1.08–2.28; p = 0.02 vs. control] and tiotropium (RR 2.12, 95% CI: 1.22–3.67; p = 0.008 vs. control) (33), long-term results from the 4-year UPLIFT study did not confirm any increased risk with tiotropium. In UPLIFT, the risk of death, cardiovascular death, myocardial infarction or stroke was not significantly increased vs. placebo; on the contrary, examination of all serious cardiac and lower respiratory tract adverse events indicated that tiotropium reduced the risk of serious adverse events (8).

### Long-acting β_2_-agonists

Long-acting β_2_-agonists, such as salmeterol and formoterol, induce bronchodilation by acting on the β_2_-adrenergic receptors located in the smooth muscle of the airways (Figure 2; 14,34). LABAs have a greater duration of effect than short-acting bronchodilators (more than 12 h vs. 4–6 h), with no loss of effect with long-term use (20,21,35). For example, studies of formoterol report prolonged and rapid bronchodilation with an onset of action at around 5 min, which is comparable with salbutamol and faster than salmeterol and which reaches peak bronchodilation within 1 h (21,36–39). The variations in speed of action between the agents may be because of the differences in mode of action; whereas salmeterol acts as a partial agonist of the β_2_-adrenergic receptor, formoterol is a full agonist (40). Evidence suggests that LABA monotherapy also results in greater improvement of symptoms, fewer exacerbations, reduced rescue use and improved overall health status compared with short-acting bronchodilator therapy (20,21,41). In a randomised controlled trial comparing 12 weeks’ administration of formoterol and the short-acting anticholinergic ipratropium, formoterol significantly increased the area under the curve for FEV_1_ compared with ipratropium (p < 0.025) (20). Formoterol also significantly improved all symptoms (all p ≤ 0.007) and quality of life, as assessed using the St George’s Respiratory Questionnaire (SGRQ) (p < 0.01 for total scores), and reduced the proportion of ‘bad days’ (defined as days with at least two individual symptom scores of two or more and/or a reduction in peak expiratory flow from baseline of more than 20%; p ≤ 0.001) and use of rescue medication (p < 0.001) compared with placebo, whereas ipratropium did not show a significant effect (20).

Inhaled LABA monotherapies are generally well tolerated and adverse events tend to be dose related (1,42). The increased risk of asthma mortality observed in the large Salmeterol Multicentre Asthma Research Trial has not been paralleled by an observed increased risk of COPD mortality in COPD trials involving LABAs. Rather, the Towards a Revolution in COPD Health (TORCH) study, which compared salmeterol/fluticasone with placebo, salmeterol alone or fluticasone alone, reported numerical reductions in mortality in both salmeterol arms compared with the placebo and ICS arms, although these reductions did not reach statistical significance (43). Unlike earlier large randomised controlled trials, which included only patients with GOLD stage III–IV COPD, TORCH included patients with mild airflow obstruction (equivalent to GOLD stage II). In the last few years, many large studies have included stage II COPD patients, although the percentage of subjects included at each level of severity (stages II–IV) has varied between studies.

### Use of LABAs and long-acting anticholinergics in combination

Given the different mechanisms and durations of action of LABAs and long-acting anticholinergics, combining agents from these two classes of long-acting bronchodilator may have the potential to provide better bronchodilation than the individual agents. Indeed, a number of randomised studies have reported complementary effects in trials using combination treatment with a LABA and tiotropium. Cazzola et al. reported that formoterol in combination with tiotropium elicited a significantly faster onset of action than tiotropium alone (but not formoterol alone) according to change in FEV_1_ 10 min after inhalation (p = 0.016) and showed a trend for greater maximum bronchodilation according to measures of FEV_1_ performed over 24 h (44). Similar effects were subsequently shown for salmeterol in combination with tiotropium (45). Addition of formoterol to tiotropium has also been shown to significantly reduce the use of rescue medications compared with tiotropium or formoterol alone and to be well tolerated (46,47). Recent data suggest such a combination may offer benefits for lung function that are greater than those with salmeterol plus an ICS. A 6-week, multi-centre, randomised, double-blind study compared formoterol plus tiotropium with salmeterol plus fluticasone in more than 600 patients with moderate COPD (48). Formoterol plus tiotropium produced significantly greater improvements in 12-h lung function profiles, indicated by the area under the curve for FEV_1_ (mean difference 78 ml, p = 0.0006) and forced vital capacity (FVC) (mean difference 173 ml, p = 0.0001), compared with salmeterol plus fluticasone. These data suggest that further studies investigating the benefits of combining tiotropium and LABAs are warranted.

## Can addition of ICS improve patient outcomes?

Inhaled corticosteroids exert a range of effects in the airways that suggest their utility in COPD (49). However, unlike the large body of evidence and widespread consensus that exists for bronchodilators as the mainstay of therapy in COPD, there are conflicting results as to the benefits of ICS therapy in COPD. A number of large randomised controlled trials have suggested that ICS monotherapy may not modify the natural history of COPD (50–52). While some small improvements in FEV_1_ have been reported, ICS monotherapy appears to have no significant effect on long-term decline in FEV_1_ in patients with mild to moderately severe COPD. However, data from a wide range of studies, meta-analyses and reviews have shown that ICS decreases the risk of exacerbations, slows the rate of decline of health-related quality of life and tends to improve survival (50–60). In particular, the Inhaled Steroids in Obstructive Lung Disease study, a UK trial in more than 700 people with moderate-to-severe COPD, reported a 25% decrease in acute exacerbations with fluticasone vs. placebo (p = 0.026; 52) that was shown to be independently associated with prevention of deterioration in health status according to SGRQ (61). Theoretically, a favourable impact of ICS on outcomes in COPD could arise from modification of the inflammatory response, but it could also occur via effects in upregulating the β_2_-adrenergic receptor (62), thus maximising the effect of β_2_-agonists.

As a result of these findings and the continual emergence of clinical data showing the benefits of combining ICS with LABA (discussed below), guidelines recommend ICS for regular treatment in combination with a long-acting bronchodilator for patients with severe COPD (FEV_1_ < 50% predicted) and repeated acute exacerbations requiring treatment with antibiotics or oral corticosteroids (Figure 1; 1,12). The reported synergistic action of ICS combined with β_2_-agonists may yet support early utilisation of ICS in mild or moderate COPD. The results of TORCH and UPLIFT, which included patients with moderate (stage II) COPD (46% of the population in UPLIFT), appear to support hypothetical benefits of ICS in milder COPD (8,43). Unpublished subgroup analyses of patients with moderate COPD in these two studies have suggested an effect on exacerbation rate and other outcome parameters (personal communication). However, further work is necessary to provide additional detail on the mode of action and the clinical effects, if any, of ICS in mild and moderate COPD.

Short-term side effects reported with ICS monotherapy include oral candidiasis and hoarseness (55,63). More serious adverse events are rare, although long-term effects do require further investigation. The impact of long-term ICS therapy on bone mineral density has been difficult to evaluate because of confounding factors, but recent studies suggest no association with an increased risk of bone mineral density or fracture (64,65). An increased incidence of pneumonia has been observed in some clinical studies using ICS therapy, either as monotherapy or in combination with a LABA (43,66–68). This finding has raised some concerns but whether this relationship applies to all ICS therapies remains uncertain as most of the data are heavily influenced by fluticasone and further investigation is required.

### Long-acting anticholinergics plus ICS

Very few studies published to date have been designed specifically to evaluate the effect of ICS and tiotropium combinations on clinical outcomes and this is an area that warrants future study. One study in Korea randomised 100 patients with COPD to receive tiotropium with or without the addition of low-dose budesonide for 6 weeks (69). While changes in trough FEV_1_ and the use of rescue medication were similar between the two groups, the tiotropium/budesonide combination was associated with a significant improvement in health status according to SGRQ total score (p = 0.003). In a second recent Korean study, Choi et al. (70) reported improvements in dyspnoea and lung function over 6 months of treatment with tiotropium and ICS combination therapy in patients with COPD and chronic obstructive bronchial asthma as well as a reduction in the frequency of exacerbations.

### ICS/LABA

**Figure 3:**
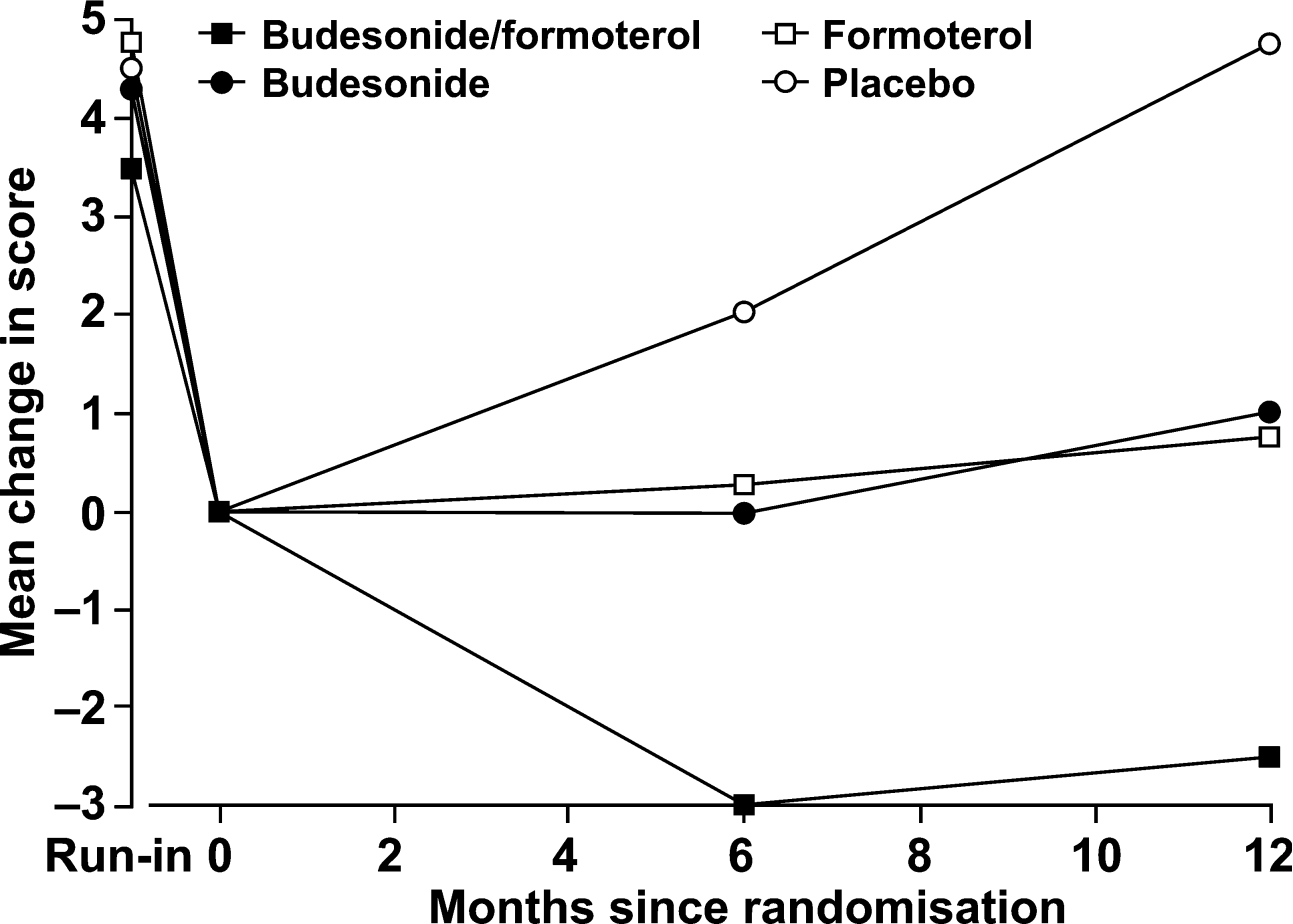
Improvements in health-related quality of life with budesonide/formoterol (71). Time course of the change in St George’s Respiratory Questionnaire (SGRQ) total scores relative to first attendance at clinic visits, in patients with moderate severe COPD. Treatment with budesonide/formoterol and the individual monotherapies improved (reduced) SGRQ total scores vs. placebo, with the greatest improvement occurring with budesonide/formoterol. Differences at 12 months were −7.5 (p < 0.001), −3.0 (p < 0.05) and −4.1 (p < 0.01) vs. placebo for budesonide/formoterol, budesonide alone and formoterol alone respectively. Reprinted from Ref. (71). Copyright © (2003), with permission from *European Respiratory Society Journals*

Combination therapy with an ICS and a LABA is recommended and widely used for patients with more severe COPD. The value of this combination and its potential to address multiple components of the disease has been established in large clinical trials. In particular, several studies have shown that treatment with the ICS/LABA combination therapies budesonide/formoterol or salmeterol/fluticasone improve lung function and exacerbations compared with placebo or LABA alone (Table 2; 15,16,43,71). One pivotal study demonstrating long-term improvements in lung function was carried out by Calverley et al. (71) who randomised more than 1000 people to receive budesonide/formoterol, budesonide alone, formoterol alone or placebo for 12 months. Initial improvements in FEV_1_ were maintained throughout the study with budesonide/formoterol, while, in contrast, FEV_1_ declined greatly and rapidly with all other treatments. The difference in percentage decline from baseline was significant with budesonide/formoterol compared with placebo (14%), budesonide (11%) and formoterol (5%) (p < 0.005). Calverley et al. (71) also reported a prolonged time to first exacerbation with budesonide/formoterol compared with all other treatments (all p < 0.05). Studies have also documented clinically meaningful improvements in health status with ICS/LABA therapy (16,71,72) and clinically meaningful improvements in patients’ health-related quality of life with budesonide/formoterol therapy, according to the SGRQ (71). Budesonide/formoterol provided a sustained reduction in SGRQ score, which after 12 months of treatment was reduced by 7.5 compared with placebo (p < 0.001); a reduction in score of 4 is considered a clinically relevant improvement (Figure 3). The improvement was also superior to that seen with formoterol and budesonide alone.

**Table 2:** Summary of results from studies on ICS/LABA combinations

					ICS/LABA vs. comparator at study end				
References	Study size	Study duration	COPD severity	ICS/ LABA dose	FEV_1_	Rate of severe exacerbations	Symptoms	Health-related quality of life	Other notes
**Budesonide/formoterol**									
Szafranski et al. (16)	812	12 months	Moderate to severe	160/4.5 μg 2 inhalation bid	+15% vs. PBO‡ +9% vs. BUD‡	−24% vs. PBO* −23% vs. FORM*	*Total symptom score* −0.21 vs. PBO‡ −0.13 vs. BUD*Breathlessness −0.36 vs. PBO‡	*SGRQ total score* –3.9 vs. PBO†	–
Calverley et al. (71)	1022	12 months	Moderate to severe	320/9 μg bid	+14% vs. PBO‡ +5% vs. FORM† +11% vs. BUD‡	−24% vs. PBO* −26% vs. FORM*	*Total score* −0.56 vs. PBO† −0.26 vs. BUD (ns) −0.02 vs. FORM (ns) *Breathlessness* −0.21 vs. PBO‡ −0.12 vs. BUD*	*SGRQ total score* Significantlyimproved vs. BUD†,FORM* and PBO‡ −7.5 vs. PBO‡	–
Bathoorn et al. (73)	45	14 days	Moderateto mild	320/9 μg 4 times daily	+125 ml vs.PBO (ns)	–	Reduced total symptom scorevs. PBO*	–	Sputum eosinophils −57% vs. +24% PBO*
**Salmeterol/fluticasone**									
Calverley et al. (15)	1465	12 months	Moderate to severe	50/500 μg bid	+133 ml vs. PBO§ +73 ml vs. S§ +95 ml vs. F§	−25% vs. PBO§	*Breathlessness* −0.19 vs. PBO‡ −0.12 vs. S† −0.11 vs. F*Cough −0.09 vs. PBO*	*SGRQ total score* −2.2 vs. PBO‡ −1.4 vs. F*	Reliever use Significantly reduced vs. PBO, F and S‡ Night-time awakenings Reduced vs. PBO, S*
Calverley et al. (43)	6112	3 years	Moderate to severe	50/500 μg bid	–	−25% vs. PBO‡ −14% vs. S† −9% vs. F†	–	*SGRQ total score* −3.1 vs. PBO‡ −2.2 vs. S‡ −1.2 vs. F†	Mortality −17.5% vs. PBO(p = 0.052)
Mahler et al. (21)	691	24 weeks	Moderate to severe	50/500 μg bid	+159 ml vs. PBO* +54 ml vs. F* +67 ml vs. S*	–	*Dyspnoea index score* +1.7 vs. PBO‡ +1.2 vs. S‡ +0.8 vs. F*	CRDQ +5.0 vs. PBO† +6.2 vs. F*	Reliever use Improvement vs. PBO, F* Night-time awakenings Improvement vs. PBO*

* p < 0.05;

† p < 0.01;

‡ p < 0.001;

§ p < 0.0001. bid, twice daily;

BUD, budesonide; COPD, chronic obstructive pulmonary disease; CRDQ, chronic respiratory disease questionnaire; F, fluticasone; FEV_1_, forced expiratory volume in 1 s; FORM, formoterol; ICS, inhaled corticosteroid; LABA, long-acting β_2_-agonist; ns, non-significant; PBO, placebo; S, salmeterol; SGRQ, St George’s respiratory questionnaire.

Notably, emerging data have supported an anti-inflammatory effect of budesonide/formoterol during exacerbations of COPD. In a small double-blind study comparing 14-day treatment with budesonide/formoterol or placebo in patients experiencing a mild to moderate COPD exacerbation, sputum eosinophils were significantly reduced by budesonide/formoterol (−57%) but increased with placebo (+24%) (p = 0.01; 73). Total symptom scores also improved compared with placebo (p = 0.01).

**Figure 4:**
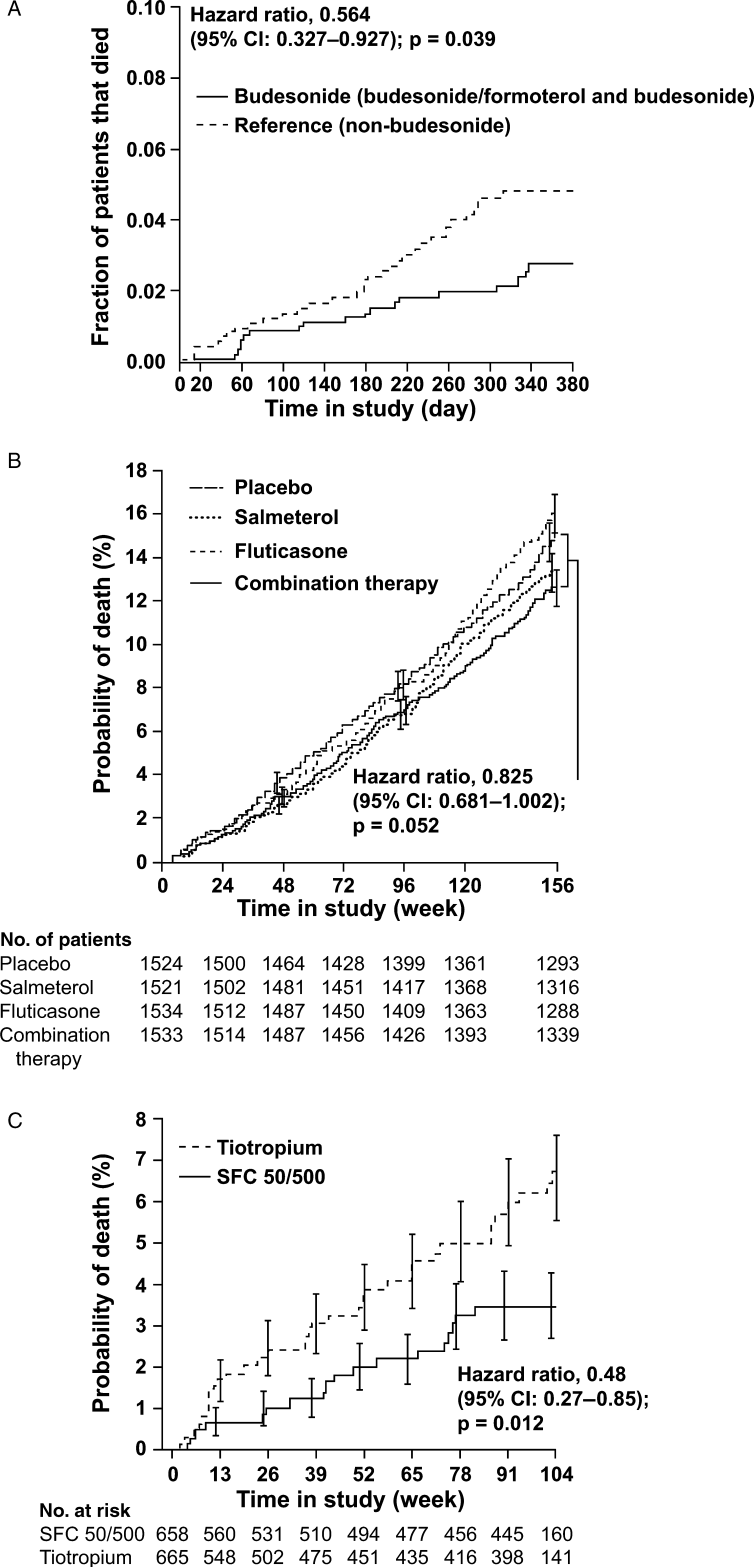
Kaplan–Meier curves for mortality. Kaplan–Meier curves for mortality for budesonide-containing therapies (budesonide alone or budesonide/formoterol) vs. (A) reference (non-budesonide) groups (72), (B) salmeterol/fluticasone vs. placebo (43) and (C) salmeterol/fluticasone (SFC) vs. tiotropium (66). (A) Reprinted from Ref. (72). Copyright © (2008), with permission from *Respiratory Medicine*. (B) Reprinted from the Ref. (43). Copyright © (2007) Massachusetts Medical Society. All rights reserved. (C) Reprinted from Ref. (66). *Official Journal of the American Thoracic Society*© American Thoracic Society

Evidence suggests that ICS/LABA combinations may have the potential to reduce mortality in COPD (Figure 4; 43,66,72). However, the large 3-year, randomised, double-blind, global mortality study TORCH (in patients with GOLD stage II–III COPD), did not demonstrate a significant benefit with regard to mortality (43). A trend towards reduced all-cause mortality (the primary end-point) of 17.5% was reported with salmeterol/fluticasone vs. placebo (p = 0.052; Figure 4B). The monotherapies (salmeterol and fluticasone) had no impact on survival. The lack of significant effect with the combination may have been related to an underpowering of the study arising from fewer deaths than anticipated in the placebo arm and a higher rate of dropout in the placebo arm. A *post hoc* analysis of the TORCH study indicated that the ICS/LABA combination had slowed the progression of COPD in this patient population (74). Compared with placebo, salmeterol/fluticasone reduced the rate of decline of FEV_1_ by 16 ml/year (p < 0.001) – a greater difference than observed with the individual agents. A reduction in mortality has also been reported with budesonide/formoterol: retrospective pooled data from two large 1-year studies revealed that budesonide, either alone or in combination with formoterol, significantly reduced the risk of death from any cause compared with formoterol or placebo maintenance therapy (Figure 4A; 72). The finding that combination ICS/LABA therapy may have a greater effect on mortality than the individual agents alone is supported by retrospective data (75,76). These include a retrospective analysis of the UK General Practice Research Database that looked at prognosis in the year after a first hospitalisation for COPD (75). The combined risk of rehospitalisation or death was significantly reduced by 41% in users of combined ICS/LABA therapy compared with a control group using short-acting bronchodilators only, but was only 16% in those prescribed ICS alone and 10% with LABAs alone (all p < 0.05). In addition, a recent US observational study reported a 22% reduction in the risk for cardiovascular death, combined cardiovascular and respiratory death and all-cause mortality in patients exposed to ICS/LABA (32).

Data also suggest that mortality may be lower with ICS/LABA compared with tiotropium alone – a significant finding given the unclear risks of mortality with tiotropium alone. The large, 2-year Investigating New Standards for Prophylaxis in Reduction of Exacerbations (INSPIRE) study was the first study to compare an ICS/LABA combination (salmeterol/fluticasone) with a long-acting bronchodilator (tiotropium) head-to-head in severe to very severe COPD (66). The study revealed a significant 3% absolute reduction in mortality with salmeterol/fluticasone compared with tiotropium alone (p = 0.032; Figure 4C) despite not being powered for mortality. The primary end-point, the overall rate of exacerbations, was not significantly different between the two treatment arms. While exacerbations requiring oral corticosteroids were less frequent with salmeterol/fluticasone vs. tiotropium (p = 0.039), exacerbations requiring antibiotics and hospitalisations were more frequent with salmeterol/fluticasone vs. tiotropium (p = 0.028 and p = 0.085 respectively). The same study reported a significantly lower SGRQ total score with salmeterol/fluticasone than with tiotropium (−2.1 units, 95% CI: −4.0 to −0.1; p = 0.038), but this did not reach the minimal clinically important difference of four units. It is important to bear in mind a number of important limitations in the INSPIRE study, including a significantly higher drop-out rate in the tiotropium arm (p = 0.005). To date, budesonide/formoterol has not been directly compared with tiotropium in COPD.

## ICS/LABA in addition to tiotropium

**Figure 5:**
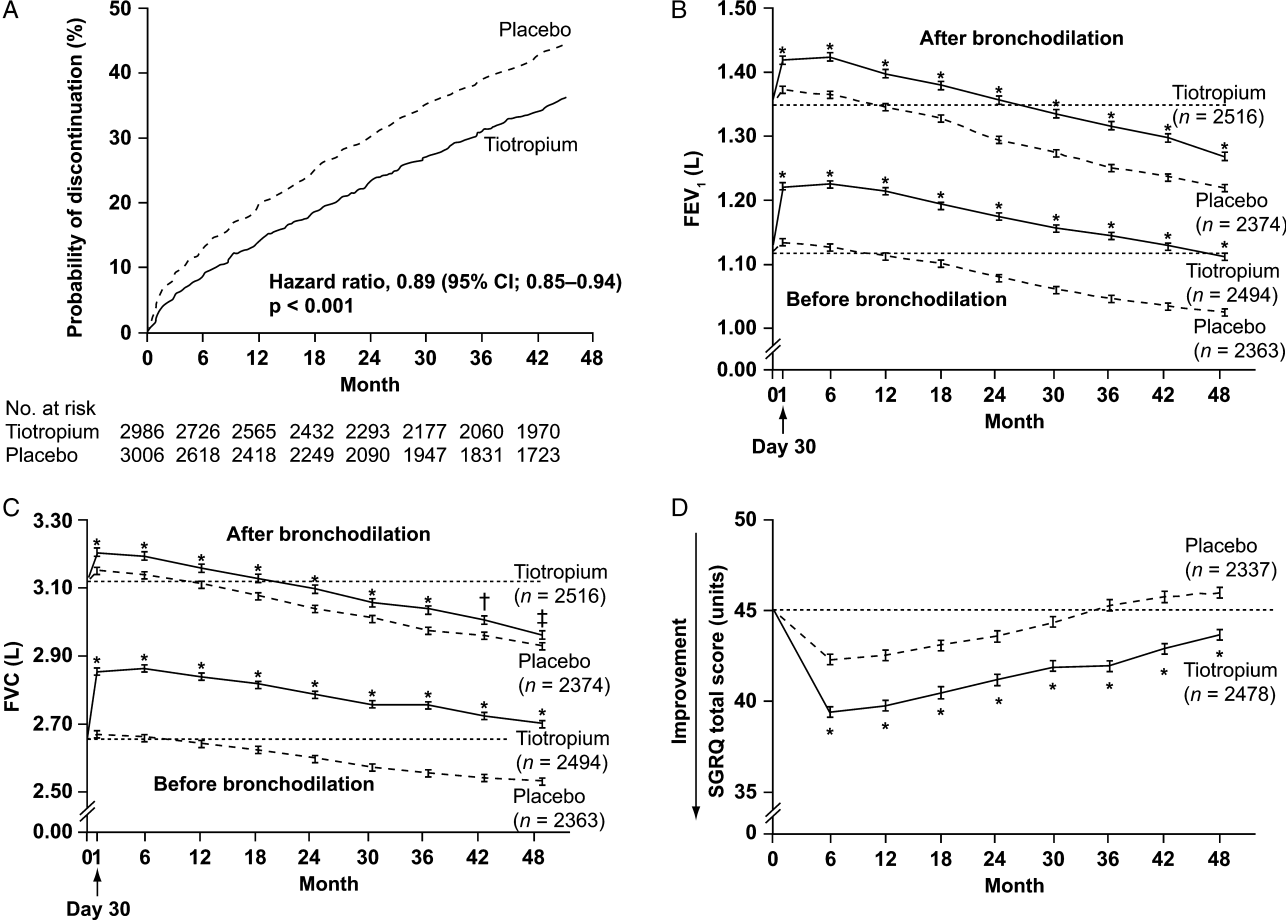
Clinical benefits of adding tiotropium to other respiratory medications (8). (A) Probability of treatment discontinuation, (B) mean forced expiratory volume in 1 s (FEV_1_) and (C) forced vital capacity (FVC) before and after bronchodilation and (D) scores for health-related quality of life in patients randomised to receive tiotropium (18 μg once daily) plus any other COPD medication apart from anticholinergics vs. placebo. Bars represent standard errors and horizontal dashed lines represent baseline levels. *p < 0.001; †p = 0.002; ‡p = 0.04. Reprinted from Ref. (8). Copyright © (2008) Massachusetts Medical Society. All rights reserved.

The efficacy of combining tiotropium with an ICS/LABA is of increasing interest given that LABAs and long-acting anticholinergics have distinct yet complementary mechanisms of action (Figure 2). The effect of combining tiotropium with other COPD medications has already been the subject of a number of studies (Table 3; 8,10,11,77). For example, in a large veterans administration trial, the addition of tiotropium to other COPD medications reduced the rate of exacerbations by 19% (p = 0.031) vs. placebo in patients with moderate to severe COPD and showed a non-significant reduction in COPD-related hospitalisations (77). However, the study period was relatively short and the beneficial effects were modest. The 4-year UPLIFT trial provided additional support for these trends. In this study, which enrolled patients with moderate to very severe COPD (> 40% had stage II COPD), tiotropium used with any prescribed COPD medication other than another inhaled anticholinergic significantly improved health-related quality of life, rate of exacerbations and lung function vs. placebo (Figure 5; 8). However, the rate of decline in FEV_1_ (primary end-point) did not improve. There were a number of limitations to UPLIFT. As discussed earlier, concomitant therapies could include other respiratory medications except anticholinergics (Table 1). In addition, there was a higher rate of discontinuation in the placebo group (44.6% vs. 36.2%; p < 0.001) and these patients had a greater rate of decline in postbronchodilator FEV_1_ compared with those who completed the study. Thus, those in the placebo group who completed the study may represent ‘healthy survivors’.

**Table 3:** Summary of results from recent studies on ICS/LABA + tiotropium combinations

				ICS/LABA + TIO (or any medication) vs. comparator at study end		
References	Study size (N)	Study duration	COPD severity	FEV_1_	Severe exacerbations (% reduction)	Symptom control
**Any COPD medication + tiotropium (18 μg once daily) vs. PBO**						
Niewoehner et al. (77)	1829 (99% men)	6 month	Moderate to severe	Increase in FEV_1_‡	−19% vs. PBO*	–
Tashkin et al. (8)	5993	4 years	Moderate to severe	Rate of decline: ns	−14% Leading to hospitalisation‡	SGRQ total score +2.7‡
**ICS/LABA (salmeterol/fluticasone or budesonide/formoterol) + tiotropium vs. ICS/LABA**						
Perng et al. (11)	46	1 month with TIO	Severe	Increase in FEV_1_‡	–	SGRQ total score +6.5‡
**Salmeterol/fluticasone + tiotropium vs. tiotropium + PBO**						
Aaron et al. (10)	449	1 year	Moderate to severe	+0.027 vs.TIO + PBO*	−2.8% vs. TIO+ PBO (ns)	SGRQ total score +4.1 vs. TIO + PBO*

*p < 0.05; †p < 0.01; ‡p < 0.001. COPD, chronic obstructive pulmonary disease; FEV_1_, forced expiratory volume in 1 s; ICS, inhaled corticosteroid; LABA, long-acting β_2_-agonist; ns, non-significant; PBO, placebo; TIO, tiotropium; SGRQ, St George’s respiratory questionnaire.

While the aforementioned studies combined tiotropium with any other concomitant COPD medication (apart from anticholinergics) further studies have more specifically reported that triple therapy with tiotropium combined with an ICS/LABA combination provides clinical benefits in lung function and improved quality of life. Perng et al. (11) showed that in patients with severe COPD treated with ICS/LABA therapy (57% of patients with salmeterol/fluticasone, 43% with budesonide/formoterol), adding tiotropium for 1 month significantly improved lung function according to FVC, FEV_1_ and inspiratory capacity. After tiotropium withdrawal, FVC, FEV_1_ and inspiratory capacity decreased markedly. Addition of tiotropium also led to significant improvements in SGRQ total scores (p < 0.001). These findings are supported by data published by Aaron et al. (10) which showed that, after 1 year, salmeterol/fluticasone plus tiotropium improved lung function (p = 0.049), quality of life (improvement in SGRQ > 4 points vs. tiotropium alone; p = 0.01) and hospitalisation rates for exacerbations (95% CI: 0.33–0.86) compared with tiotropium alone in patients with moderate to severe COPD. By contrast, similar significant benefits with regard to hospitalisation rates were not seen for therapy with tiotropium plus salmeterol. In this study, the combination salmeterol/fluticasone and tiotropium compared with tiotropium alone had no significant effect on the rate of exacerbations, although this effect may have arisen through underpowering of the study and the small number of exacerbations that occurred. It is clear that, while the potential appears substantial, more research is needed into the specific effects of adding tiotropium to ICS/LABA.

## Treatment strategies in advanced COPD

Specific non-pharmacological strategies assume an important role in management of advanced COPD. Oxygen therapy is one of the principal non-pharmacological treatments for patients with very severe (stage IV), hypoxic COPD (1) and has been shown to improve survival (78). The benefits of intermittent non-invasive ventilation in patients with hypercapnic respiratory failure are less clear (79), and a multi-centre study is underway in Germany with the aim of providing new data. Controlled data on pharmacological interventions are generally not available for patients with respiratory failure. However, as exacerbation rate is the key prognostic factor in these patients, it may be argued that any pharmacological therapy with evidence to support reductions in exacerbations continues to play a role in optimised management.

## Investigating budesonide/formoterol added to tiotropium: the CLIMB study

**Figure 6:**
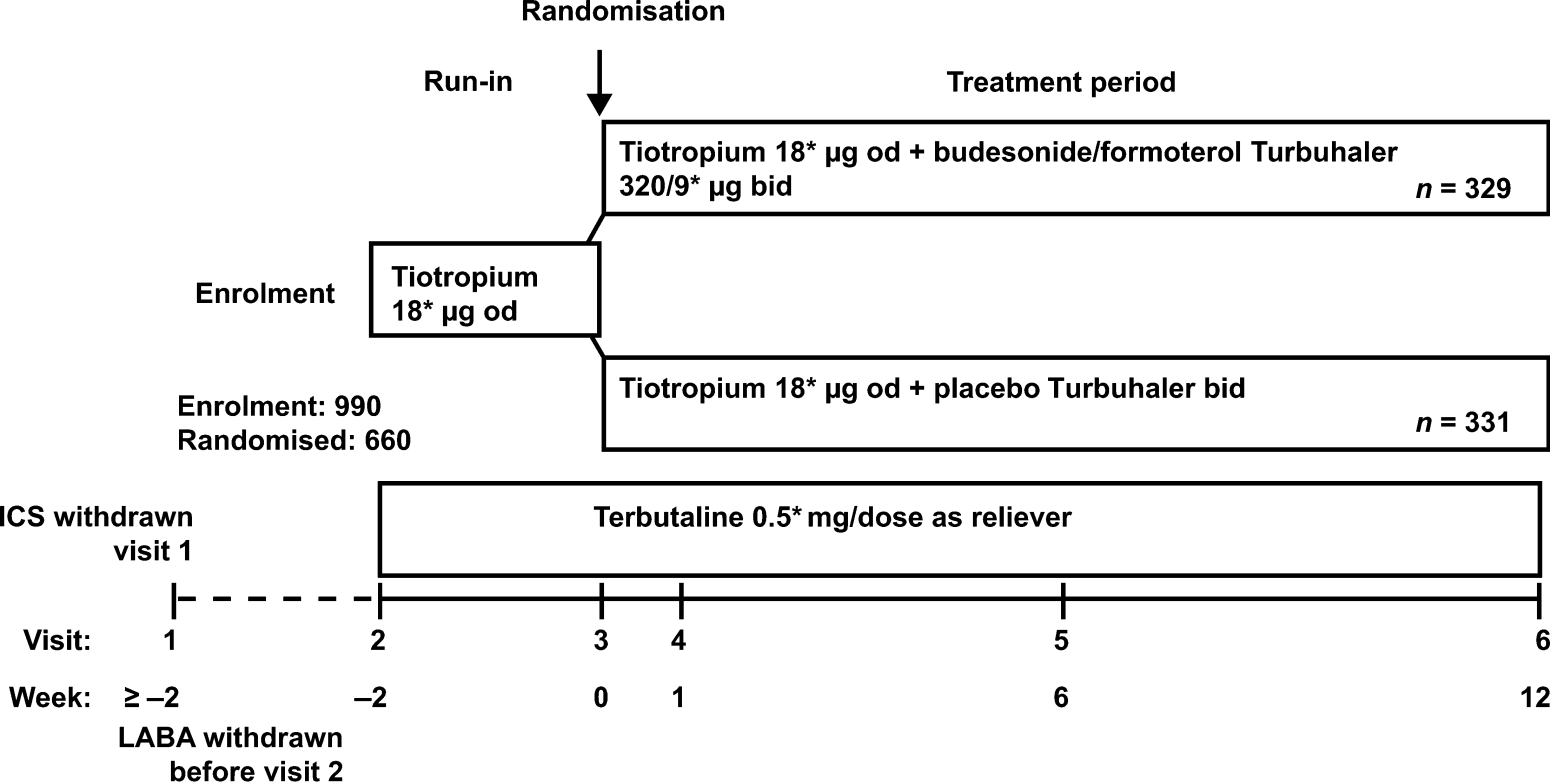
CLIMB study design. ICS, inhaled corticosteroid; LABA, long-acting β_2_-agonist. *All doses expressed as metered doses

As yet, no study has specifically reported the treatment benefits of triple therapy combining tiotropium with budesonide/formoterol in patients with COPD. To meet this need, the 3-month CLIMB study (Evaluation of Efficacy and Safety of Symbicort as an Add-on Treatment to Spiriva in Patients With Severe COPD) was initiated in May 2007 (ClinicalTrials.gov Identifier: NCT00496470; http://clinicaltrials.gov/ct2/show/NCT00496470). CLIMB has enrolled 990 patients from nine countries with GOLD stage II–IV COPD (Figure 6). Patients are aged ≥ 40 years, have had a clinical diagnosis of COPD with symptoms for at least 2 years and experienced at least one COPD exacerbation in the previous 12 months, are current or previous smokers with a smoking history of ≥ 10 pack years and have an FEV_1_ ≤ 50% of predicted normal value and FEV_1_/FVC < 70% prebronchodilator. The primary efficacy outcome measure is the change in predose FEV_1_ from baseline, as assessed using spirometry at clinic visits. Other important variables assessed include time to first exacerbation and frequency of exacerbations, health-related quality of life according to SGRQ and morning symptoms and activities. Any effect of budesonide/formoterol added to tiotropium on improving morning activities and morning lung function will be notable in light of evidence suggesting COPD patients struggle to undertake morning activities (6).

A number of design features in CLIMB may eliminate some of the limitations affecting the previous studies of triple therapy involving ICS/LABA and tiotropium. Notably, the CLIMB protocol does not permit the addition of concomitant maintenance therapies, whereas studies such as UPLIFT have allowed concomitant therapy other than anticholinergics, thus potentially confounding results. In addition, CLIMB includes a phase withdrawal during the run-in period which allows patients who used ICS before the study to withdraw before randomisation if dissatisfied with the management of their symptoms with tiotropium alone. This, together with the use of active symptomatic treatment (all patients receive tiotropium after study enrolment), should overcome differential dropout which can modify the study outcome. Differential dropout from a study favours the less effective treatment, resulting in a ‘healthy survivor effect’, particularly in placebo-treated patients who receive no symptomatic treatment, reducing the difference between groups for the study outcome; this has been a limitation of some of the previous studies discussed (8,10). The first results from CLIMB are expected to report in mid-2009.

## Conclusions

Chronic obstructive pulmonary disease is a multi-component disease; for optimal treatment, combining therapies with complementary modes of action enables multiple components of the disease to be treated. Current guidelines recommend ICS/LABA combination therapy for the management of severe COPD and several randomised clinical trials have shown the benefits of this approach. However, recent evidence suggests that triple therapy combining ICS/LABA with tiotropium can provide further clinical benefits and may improve quality of life because of the effects of their complementary modes of action in maintaining airway calibre. Further clinical evidence is required to confirm the extent of these benefits, particularly in the morning when the impact of the disease may be at its most severe. Moreover, the effect of combining tiotropium with budesonide/formoterol has yet to be directly assessed. These will be addressed by the CLIMB study.
